# Behavioural risks in male dogs with minimal lifetime exposure to gonadal hormones may complicate population-control benefits of desexing

**DOI:** 10.1371/journal.pone.0196284

**Published:** 2018-05-02

**Authors:** Paul D. McGreevy, Bethany Wilson, Melissa J. Starling, James A. Serpell

**Affiliations:** 1 Sydney School of Veterinary Science, Faculty of Science, University of Sydney, Sydney, New South Wales, Australia; 2 School of Veterinary Medicine, University of Pennsylvania, Philadelphia, Pennsylvania, United States of America; Gaziosmanpasa University, TURKEY

## Abstract

Castration of dogs is a widespread practise with clear justification in population control and knock-on benefits for animal welfare. Deleterious behavioural consequences of castration are believed to be negligible. Gonadectomy is widely recommended as part of a multi-factorial approach to prevent problems including aggression in dogs. However, the consequences of early castration on health are still being debated. The current study focused on the reported behaviour of 6,235 male dogs castrated before 520 weeks of life for reasons other than behavioural management, and calculated their percentage lifetime exposure to gonadal hormones (PLGH) as a proportion of their age at the time of being reported to the online Canine Behavioral Assessment and Research Questionnaire (C-BARQ). Forty behaviors differed between entire and castrated dogs, of which 25 were associated with PLGH and 14 with age-at-castration (AAC). Only 2 behaviours, indoor urine marking and howling when left alone, were significantly more likely in dogs with longer PLGH. In contrast, longer PLGH was associated with significantly reduced reporting of 26 (mostly unwelcome) behaviours. Of these, 8 related to fearfulness and 7 to aggression. The current data suggest that dogs’ tendency to show numerous behaviours can be influenced by the timing of castration. They indicate how dog behaviour matures when gonadal hormones are allowed to have their effect. The differences reported here between undesirable behaviours of castrated and intact dogs were in the range of 5.04% and 12.31%, suggesting that, for some dogs, partial or complete denial of puberty may reduce indoor urine-marking but have many other undesirable consequences. Veterinarians may use these data to discuss unwelcome consequences with owners of male dogs before castration.

## Introduction

Domestic dogs (*Canis lupus familiaris*) are one of the most commonly kept animals in developed countries, so it is important to understand their basic needs. Failure to meet these needs can result in behavioural problems [1]. Dog behaviour, particularly aggressive, fearful and sociable behaviour, can differ between sexes. Male dogs are reputedly more aggressive than females, and tend to predominate in bite statistics [2, 3, 4, 5, 6]. They can be bolder than female dogs, where boldness is considered a super-trait incorporating a lack of fear and increased interest in interacting with social objects [7, 8]. Entire (also known as intact) dogs of both sexes are bolder than gonadectomised (also known as neutered or desexed) dogs [8] and boldness decreases with aging [8]. If boldness predicts less fearful behaviour and more sociable behaviour with humans and dogs, it would be desirable in companion dogs, so the potential for individual boldness to change during life raises questions as to how common practices, such as gonadectomy, may contribute to problem behaviours associated with this super-trait.

There are many excellent reasons for gonadectomy that relate to control of numbers in the broader population. Gonadectomy at, or just before, sexual maturity has been common practise for decades, chiefly to avoid unwanted litters. More recently, the merits of very early gonadectomy (at less than 12 weeks) have been emphasised by those focusing on population control.

Reasons for any putative effects of gonadectomy on longevity differ in males and females [9]. In males, it is known to increase the risk of prostate cancer but the benefits may include removing the risk of testicular disease and other androgen-dependent diseases, such as perineal hernias, perineal adenomas, prostatitis and benign prostatic hyperplasia (reviewed in ref. [10]). Additionally, gonadectomised dogs face 0.1 times the odds of developing mast cell tumours [95% CI 0.1 to 0.2) than entire dogs [11]. Castrated males may also wander less in search of mating opportunities, an outcome with the additional benefits of fewer traffic accidents and unwanted litters [12]. In females, spaying was thought to reduce the risk of mammary neoplasia [13] but a recent systematic review described evidence for this association as being only weak [14].

Despite these reported benefits, gonadectomy rates remain low in some countries. For example, in a sample of dogs (n = 10,519) owned by German-speaking dog enthusiasts, only 43.1% were gonadectomised [15], while among dogs attending UK veterinary clinics (n = 148,741), 41.1% were gonadectomised [16]. By contrast, in Australia, there is evidence that 77% of male dogs and 85% of female dogs are gonadectomised [17], and in the U.S., an estimated 83% of all dogs are gonadectomised [18].

Some health benefits of gonadectomy may be offset if it compromises health in other ways. For example, spaying female golden retrievers has been associated with a 3–4 fold increase in the rate of some cancers [19]. Same-breed castrated males showed only minor increases in risk of cancers. In another study, castrated males of many breeds had a higher risk of diabetes mellitus than entire males, whereas spaying was not associated with an increased risk in females [20]. Gonadectomy has also been associated with increased bone length [21], which may, in part, explain why studies focused on osteosarcoma found twice the prevalence in gonadectomised dogs than in entire dogs of several breeds [22] and an increase of 3–4 times in Rottweilers castrated before 1 year of age [23]. Similarly, studies of golden retrievers [24] and German shepherd dogs [25] have shown that early neutering can significantly increase the prevalence of joint disorders, such as hip dysplasia.

Veterinarians may advise the castration of adult male dogs that have presented with aggressive behaviour, notably towards the people or other dogs in the household. Despite the historic propensity for veterinarians to recommend castration to treat problem behaviours, the effect of sex and gonadal status on dog behaviour has been assessed in only a few studies. In a recent study of free-roaming male dogs, surgically castrated dogs showed no less sexual activity or aggression 6 months after surgery than before [26]. Meanwhile, tests of spatial learning, memory and reversal learning tasks using a T-maze showed that 81% of entire females successfully completed the whole procedure compared to 56% of spayed females, 62% of entire males and 50% of castrated males [27]. Reports from owners of 209 male and 382 female dogs revealed that, after gonadectomy, male dogs showed behavioural changes more often and more distinctly than female dogs [28]. For 49 of 80 (61%) aggressive male dogs and 25 of 47 (53%) female dogs, these reports described dogs becoming more gentle after gonadectomy [29]. That said, 10 females became aggressive after being gonadectomy. Notably, increased voluntary food intake emerged in 42% of male dogs and 32% of female dogs. Weight gain and resultant lethargy are expected in many gonadectomised dogs [28] and owners should be counselled on how to prevent these outcomes [12]. Calorie-restriction may increase the perceived value of rations to gonadectomised dogs and lead to food-guarding [12], but associations between gonadectomy and food-related aggression have been challenged [1]. In contrast, male dogs have shown higher levels of separation-related distress (SRD) than females [1, 29] and entire dogs have shown more SRD than castrated dogs [1]. Gonadectomised dogs of both sexes have been shown to be more aggressive, fearful, and excitable than entire dogs [21]. Similarly, they have been reported to be less trainable than entire dogs [21], but a study with a focus on Shetland sheepdogs reported castrated males to be more trainable than entire males [30].

An extensive literature review, published in 2010, concluded that evidence from intervention studies failed to demonstrate clear behavioural outcomes after surgical castration in male dogs [31]. By contrast, a recent systematic review of the role of gender and gonadal status in reported dog bites directed to humans drew data from 6 studies (total study population n = 53,763 dogs) and found that castration is associated with a reduced risk of dog bite [32]. However, the authors emphasised the absence of causality and acknowledged that neither age-at-castration (AAC) nor reasons for castration had been recorded for this analysis.

In summary, the effects of castration on dog behaviour have not been exhaustively investigated, and relevant studies sometimes produce conflicting results. The current study examines data from the owners of male dogs to explore relationships between various canine behavioural attributes, as reported through C-BARQ, and castration considering both AAC and percentage lifetime exposure to gonadal hormones (PLGH) as factors. Clearly, AAC and PLGH are related but the latter indicates how long the dog has lived with gonadal hormones until the age of castration, and can reveal the influence of gonadal hormones on behaviour after puberty, reflecting the role of circulating hormones in the normal behavioural biology of dogs.

## Method

### C-BARQ

The C-BARQ is an owner-completed survey instrument designed to provide quantitative assessments of a wide array of behavioural characteristics of dogs. It consists of 100 items that ask respondents to indicate, using a series of 5-point ordinal rating scales, their dogs’ typical responses to a variety of everyday situations during the recent past. The scales rate either the intensity (aggression, fear and excitability subscales) or frequency (all remaining subscales and miscellaneous items) of the behaviours, with a score of 0 indicating the absence of the behaviour and a score of 4 indicating the most intense or frequent form of the behaviour [33, 34]. The online C-BARQ survey (http://vetapps.vet.upenn.edu/cbarq/) was reviewed by the University of Pennsylvania Institutional Review Board and determined to be exempt from IRB approval because no personal identifying information other than email address is collected from dog owners during survey completion. The requirement for informed consent was also waived. Participants are not actively recruited to complete the C-BARQ. Beginning in 2006, the original online survey was advertised via an article in the newsmagazine of the School of Veterinary Medicine at the University of Pennsylvania (http://www.vet.upenn.edu/bellwether/v64/article10.shtml), and by notices sent to Philadelphia-area veterinary clinics and the top 20 USA breed clubs based on AKC registrations. Since that time, information about the survey has been disseminated via word-of-mouth among dog owners who visit the website and complete the questionnaire of their own volition.

The C-BARQ currently comprises 14 behavioural factors or subscales, and a further 22 miscellaneous stand-alone items. Higher scores are generally less favourable for all items and subscales with the exception of ‘trainability’, for which higher scores are more desirable. For the purposes of the current study, we drew data only on male dogs castrated before 520 weeks (10 years) of age.

C-BARQ asks respondents to specify the reasons for castration. We accepted data on dogs castrated at least two weeks prior to C-BARQ evaluation for the following reasons: required by breeder/shelter (n = 3031), birth control (n = 2444), prevention of health problems (n = 622), and correction of health problems (n = 138). We excluded dogs (n = 2712) castrated for the following reasons: to correct a behaviour problem (n = 537), to prevent a behaviour problem (n = 638), recommended by vet (n = 685) and unknown (n = 852).

### Candidate attribute selection

The C-BARQ data included ordinal item-scores for 100 behavioural attributes, from which 14 behavioural subscales were extracted by factor analysis [33]. Because linear models make assumptions based on a continuously varying underlying trait, rather than a composite quasi-continuous trait assembled from ordinal scorings, analysis was undertaken on the ordinal scores directly using models better suited to ordinal data. To better meet the assumptions of the underlying models, analysis was undertaken on the ordinal item scores directly.

Because of the large numbers of attributes involved, candidate attributes selection was undertaken prior to the primary analysis, to identify attributes in which there were clinically significant behavioural differences between entire and castrated dogs. C-BARQ scores from 3392 entire male dogs and 6546 male dogs castrated for eligible reasons (see above) were compared. For the 27 breeds and crosses tested, *see* S1 Table.

Each behaviour was coded as a binary variable (high/low), according to the score received in the C-BARQ and that the frequency of behaviours coded as high or low were compared between castrated and intact male dogs. For most studied behaviours, dogs were considered to have demonstrated either a low tendency for the behaviour (a score of Never) or a higher tendency for the behaviour (any other score). For behaviours “Playful, puppyish and boisterous” and “Active, energetic, always on the go”, a more moderate division of scores was applied where scores of Never, Seldom or Sometimes were considered a low tendency, and scores of Usually or Always were considered a high tendency. The more moderate division was intended to reflect the presumed desirability of moderate frequency of these behaviours. Several Trainability behaviours (“When off the leash, returns immediately when called”, “Obeys the ‘sit’ command immediately”, “Obeys the ‘stay’ command immediately”, “Seems to attend/listen closely to everything you say or do” and “Will ‘fetch’ or attempt to fetch sticks, balls, or objects”) were also classified differently, with Usually or Always being grouped together to represent a high tendency. This was decided by the authors because ‘Always’ was subjectively considered to be too stringent a standard to be truly informative for these behaviours (i.e., a dog that ‘Usually’ sits immediately on command would be reasonably considered to have a high tendency to obey the sit command.

The percentage of entire and castrated dogs displaying a high tendency for the behaviour for each attribute were compared. Of the 100 behaviours, 40 showed a difference of more than 5% in the proportion of high tendency dogs between the “entire” and the “castrated” cohorts.

#### PLGH

The age of the dog when it was evaluated through C-BARQ and its reported AAC were used to calculate its lifetime exposure to hormones at the time of C-BARQ evaluation using the formula: AAC/Age at time of C-BARQ evaluation x 100. It is worth noting that we have no behavioural data on the dogs’ entire lifespan. Peaks at around 24, 36 and 52 weeks are probably artefacts resulting from the automated conversion of months or years to weeks by the C-BARQ database (see S1 Fig). We combined C-BARQ responses of 3 and 4 on the severity of a behaviour scale to arrive at four gradations: 0, 1, 2, 3+. As stated above, we nominated a 5% difference in the frequency of behaviours between castrated and entire dogs as the threshold for a trait to undergo further analysis.

### Statistical analysis

#### Model age of castration

The effect of AAC on C-BARQ scores was evaluated by a cumulative link model [35] with a logit link function, using the polr() function of the MASS package of R statistical software (R Foundation for Statistical Computing, Vienna, Austria. URL https://www.R-project.org/).

This ordinal logistic regression model had the following terms:

Y ~ ACC + AgeAtEvaluation + Breed + Weight + Breed.Weight + FirstOwned + OtherDogs + Roles.

where y denotes the ordinal C-BARQ score and logit link function, AAC (weeks), Breed of dog (see above), Weight is the weight of the dog (in pounds reported at C-BARQ evaluation), Breed.Weight is an interaction term between breed and weight, AgeAtEvaluation denotes the age of the dog at C-BARQ evaluation (in weeks), FirstOwned denotes a binary variable indicating whether or not this is the owner’s first dog, Other Dogs is a binary variable indicating whether or not there are other dogs in the household, and Roles is a categorical variable indicating whether the dog is reported to be involved with breeding/showing, working roles, field trials/hunting, other sports, or none of the above.

#### Models PLGH evaluation

We used two candidate models:

*a full model*,

Y ~ PLGH + AgeAtEvaluation + PLGH.AgeAtEvaluation + Breed + Weight + Breed.Weight + FirstOwned + OtherDogs + Roles,

which includes PLGH and an interaction term between PLGH and AgeAtEvaluation; and

*a reduced model*,

Y ~ PLGH + AgeAtEvaluation + Breed + Weight + Breed.Weight + FirstOwned + OtherDogs + Roles,

which does not include this second interaction term.

After calculation of the models, the Anova() function of the “car” package for R was used to calculate Anova tables for the model effects. A Holm-Bonferroni correction for multiple comparisons was applied to the *P* values arising from models for each trait to further control the risk of Type 1 error resulting from the large number of traits (i.e. 40) being tested. Once a Holm-Bonferroni correction for multiple comparisons had been applied, the interaction of PLGH and AgeAtEvaluation was significant for only 3 of the 40 traits of interest (“excitement just before a car trip”, being “playful, puppyish and boisterous”, and being “active, energetic, always on the go”). Therefore, the reduced model was preferred and its results are discussed further.

## Results

Among the 6235 dogs castrated prior to 520 weeks of age, AAC was positively skewed with a median of 30 weeks (6.9 months), a mean of 57.1 weeks (13.2 months) and a standard deviation of 70.6 weeks (16.3 months). PLGH for these dogs was less positively skewed, with a median of 22.0%, a mean of 31.2% and a standard deviation of 26.3%.

Of the 40 behaviours that differed between entire and castrated dogs (see S2 Table), 25 were associated with PLGH and 14 with AAC. Specifically, 26 behaviours were significantly reduced for either AAC or PLGH or both (howling and marking were significantly increased, so the total number that changed was 28). For PLGH alone, 23 behaviours were reduced (howling and marking were significantly increased, so the total that changed was 25). The behaviours that decreased as PLGH increased related chiefly to fear (n = 8) and aggression (n = 7). For AAC alone, 13 were reduced (marking was significant but increased, so the total that changed was 14).

Table 1 shows that the fear-related behaviours that decreased with increasing PLGH included those shown in response to sudden or loud noises (with a difference of 11.49%; an odds ratio of 0.996 (Uncorrected 95% Confidence interval of 0.994–0.998)). This odds ratio of 0.996 means that, for every additional 1% of a dog’s pre-CBARQ life for which it was entire, the odds of showing higher levels of fear were multiplied by 0.996. This corresponds to an odds ratio of 0.963 (0.945–0.982) for every additional 10% of a dog’s pre-CBARQ life before castration and an odds ratio of 0.911 (0.868–0.956) for every additional 25%. Other fear-related behaviours that decreased with increasing PLGH related to strange or unfamiliar objects on or near the sidewalk (difference of 5.13%; OR = 0.994(0.992–0.996)). Less fear was also found with increasing PLGH when dogs were having nails clipped by a household member (12.05%; OR = 0.995(0.993–0.997)); when barked at, growled at, or lunged at by an unfamiliar dog (10.43%; OR = 0.997(0.995–0.999)); when examined/treated by a veterinarian) (9.39%; OR = 0.995(0.993–0.997)); and approached by an unfamiliar dog of similar or larger size (6.93%; OR = 0.997(0.994–0.999)).

**Table 1:** Association of timing of castration (AAC, PLGH and the interaction between PLGH and Age of evaluation) with fear- and anxiety-related responses for male dogs. An * indicates that the *P*-value is significant after the application of a Holm-Bonferroni multiple comparisons correction.

Response		ANOVA (type II) statistics		
	Age at Castration likelihood-ratio χ^2^	*P* value	PLGH (Reduced PLGH Model) Likelihood -ratio χ^2^	*P* value
**Non-social Fear**	** **			
During thunderstorms, firework displays, or similar events	3.08	0.079	5.95	0.015
When first exposed to unfamiliar situations (e.g., first car trip, first time in elevator, first visit to veterinarian, etc.)	6.74	0.009	12.36	<0.001*
In response to wind or wind-blown objects.	4.81	0.028	8.18	0.004
In response to strange or unfamiliar objects on or near the sidewalk (e.g., plastic trash bags, leaves, litter, flags flapping, etc.)	18.06	<0.001*	31.31	<0.001*
In response to sudden or loud noises (e.g., vacuum cleaner, car backfiring, road drills, objects being dropped, etc.)	3.91	0.048	14.36	<0.001*
**Touch Sensitivity**				
When having nails clipped by a household member	8.69	0.003	21.67	<0.001*
When examined/treated by a veterinarian	3.34	0.068	24.54	<0.001*
When groomed or bathed by a household member	0.20	0.652	2.29	0.131
When having his/her feet towelled by a household member	0.03	0.860	2.71	0.100
**Dog-directed Fear**	** **			
When barked at, growled at, or lunged at by an unfamiliar dog	2.01	0.156	9.86	0.002*
When approached directly by a smaller unfamiliar dog	1.64	0.200	3.35	0.067
When approached directly by an unfamiliar dog of same or larger size.	4.30	0.038	10.76	0.001*
When unfamiliar dogs visit your home	1.55	0.213	4.21	0.040
**Stranger-related Fear**	** **			
When unfamiliar persons visit your home	0.32	0.573	1.52	0.217
When approached directly by an unfamiliar adult while away from your home	0.00	0.954	1.22	0.270
When approached directly by an unfamiliar child while away from your home	0.80	0.371	9.47	0.002*
When an unfamiliar person tries to touch or pet the dog	0.25	0.617	3.45	0.063
**Separation related Problems**				
Howling when separated	5.78	0.016	10.44	0.001*

* Significant p values at the level 0.05.

Less fear was observed in association with PLGH when these dogs were first exposed to unfamiliar situations (7.13%; OR = 0.996(0.995–0.998)) and when approached directly by an unfamiliar child while away from home (6.61%; OR = 0.996(0.994–0.999)). On the downside, howling when left alone increased in association with PLGH (5.94%; OR = 1.004(1.002–1.007)).

Of the behaviours that were less frequently associated with increasing PLGH, 7 were related to aggression (see Table 2)and were observed as follows: when delivery workers approach home (8.54%; OR = 0.992(0.990–0.994)); when strangers walk past home while dog is outside (6.88%; OR = 0.993(0.991–0.995)); when joggers, cyclists, roller-bladers or skateboarders pass home while dog is outside (7.85; OR = 0.993(0.991–0.995)); when approached directly by unfamiliar female dog while being exercised on leash (10.98%; OR = 0.996(0.994–0.998)); when unfamiliar person approaches owner or another family member at home (5.87%; OR = 0.996(0.994–0.998)); toward cats, squirrels or other small animals entering yard (6.76%; OR = 0.996(0.994–0.998)); and when unfamiliar persons visit home (5.41%;—OR = 0.996(0.994–0.998)).

**Table 2:** Association of timing of castration with aggression-related responses for male dogs.

Type of aggression	ANOVA (type II) statistics			
	AACLikelihood-ratio χ^2^	*P* value	PLGH (ReducedPLGH Model) Likelihood-ratio χ^2^	*P* value
**Dog-directed**				
When approached directly by an unfamiliar female dog while being walked/exercised on a leash	2.77	0.096	13.83	<0.001*
**Stranger-directed**				
When mailmen or other delivery workers approach your home	32.93	<0.001*	68.59	<0.001*
When joggers, cyclists, roller-bladers or skateboarders pass your home when your dog is outside or in the yard	22.57	<0.001*	41.79	<0.001*
When strangers walk past your home while your dog is outside or in the yard	22.82	<0.001*	52.38	<0.001*
When an unfamiliar person approaches you or another member of your family at home	3.56	0.059	12.26	<0.001*
Toward unfamiliar persons visiting your home	3.77	0.052	14.14	<0.001*
When approached directly by an unfamiliar adult while being walked/exercised on a leash	1.97	0.161	3.37	0.066

* Significant p values at the level 0.05.

The other behaviours (see Table 3) less frequently observed in association with PLGH were: eating own or others’ faeces (6.96%; OR = 0.996(0.994–0.998)); rolling in faeces or other “smelly” substances (6.02%; OR = 0.993(0.990–0.995)); and barking persistently when alarmed or excited (6.42%; OR = 0.994(0.993–0.996)). Indoor marking increased in association with PLGH (8.68%; OR = 1.011(1.008–1.014)). However, mounting objects, furniture, or people (7.64%; OR = 0.991(0.989–0.994)), chasing animals (-5.81%; OR = 0.991 (0.988–0.993)), and excitement when doorbell rings (5.04%; OR = 0.987(0.985–0.989)) were observed less frequently.

**Table 3:** Association of timing of castration with excitability, energy and miscellaneous responses for male dogs.

Response	ANOVA (type II) statistics			
	AAC likelihood-ratio χ^2^	*P* value	PLGH (Reduced PLGH Model) Likelihood -ratio χ^2^	*P* value
**Excitability**				
Excitable just before being taken on a car trip	0	0.996	7.88	0.005
Excitable when doorbell rings	57.16	<0.001*	147.06	<0.001*
**Energy**				
Playful, puppyish, boisterous	13.93	<0.001*	1.48	0.224
Active, energetic, always on the go	11.13	0.001*	2.22	0.136
**Miscellaneous**				
Aggression toward cats, squirrels or other small animals entering your yard	4.48	0.034	16.18	<0.001*
Chases or would chase squirrels, rabbits and other small animals given the opportunity	51.61	<0.001*	77.51	<0.001*
Urinates against objects/furnishings in your home.	53.06	<0.001*	62.46	<0.001*
“Mounts” objects, furniture, or people	48.5	<0.001*	41.97	<0.001*
Licks him/herself excessively	0.15	0.695	4.43	0.035
Eats own or other animals’ droppings or faeces	6.56	0.010	13.23	<0.001*
Steals food	13.56	<0.001*	8.25	0.004
When off the leash, returns immediately when called	2.66	0.103	9.84	0.002*
Barks persistently when alarmed or excited	13.08	<0.001*	31.42	<0.001*
Will “fetch” or attempt to fetch sticks, balls, or objects	40.43	<0.001*	34.00	<0.001*
Rolls in animal droppings or other “smelly” substances	28.12	<0.001*	50.52	<0.001*

* Significant p values at the level 0.05.

On the downside, trainability decreased with PLGH specifically, when off leash, returning immediately when called (6.53%; OR = 0.997(0.994–0.999)), and “fetching” or attempting to fetch objects ((6.22%; OR = 0.994(0.992–0.996)).

AAC revealed fewer significant associations than PLGH did (see Tables 1 and 2). Only one behaviour, indoor urine marking (OR = 1.003(1.003–1.004 per week–equivalent to 1.198 (1.143–1.256) per year) was more likely in dogs castrated later in life (see S2 Table). AAC was associated with significantly reduced reporting of 13 behaviours (*see* Tables 2 and 3). Of these behaviours, 1 related to signs of fearfulness in response to strange or unfamiliar objects on or near the sidewalk (5.13%, OR = 0.998(0.997–0.999)), and another 3 related to signs of aggression. Specifically, these dogs showed less aggression when mailmen or other delivery workers approached the home (8.54%; OR = 0.998(0.997–0.999)); when joggers, cyclists, roller-bladers or skateboarders passed the home (7.85%; OR = 0.998(0.997–0.999)); and when strangers walked past the home (6.88%; OR = 0.998(0.997–0.999)).

Barking and excitement were also less likely in dogs with increased AAC. Specifically, these dogs showed less persistent barking when alarmed or excited (6.42% 0.999(0.998–0.999)) and less excitement when the doorbell rang (5.04%; OR = 0.997(0.996–0.998)). They were also less likely to “fetch” or attempt to fetch objects (6.22%; OR = 0.998(0.997–0.998)), be playful (7.42%; OR = 0.999(0.998–0.999)), and energetic (9.69%; OR = 0.999 (0.998–0.999)).

Increased AAC was associated with significantly less mounting of objects, furniture, or people (7.64%; OR = 0.996(0.995–0.997)), less coprophagia (6.96%; OR = 0.999(0.998–1.000)) and less rolling in faeces or other “smelly” substances (6.02% OR = 0.998(0.997–0.999)).

After Holm-Bonferroni correction, 12 behaviours that differed between entire and castrated dogs failed to reach significance for either PLGH, or AAC (*see* Tables 2 and 3). Of these, 9 related to fear: during thunderstorms, firework displays, or similar events; in response to wind or wind-blown objects; when groomed or bathed by a household member; when having his/her feet towelled by a member of the household; when approached directly by an unfamiliar smaller dog; when unfamiliar dogs visit the home; when unfamiliar persons visit the home; when approached directly by an unfamiliar adult while away from home; and when an unfamiliar person tries to touch or pet the dog, although many of these traits showed significance before correction for multiple comparisons. The remaining 3 behaviours related to aggression when an unfamiliar person approaches dog while being exercised on leash, the dog licking him or herself excessively, and being excitable just before a car trip.

## Discussion

The beneficial effects of gonadectomy are underpinned by the need to reduce the number of unwanted companion animals. Thousands of dogs are euthanased in shelters and pounds annually in many developed countries [36, 37, 38, 39]. However, shelters are inundated by dogs that are most commonly surrendered because they display undesirable behaviours [40]. So the current findings present the paradox that castration may reduce the numbers of unwanted dogs but may also increase the likelihood of problem behaviours that reduce the appeal of the castrated dogs and make them more vulnerable to being surrendered. Responsible pet ownership does not end with having one’s pet gonadectomised. Rearing dogs and managing them in ways that meet their behavioural needs and enrich the bonds they share with their owners must be given priority as a form of preventative care. The challenges that owners face and the role of unwanted behaviours in jeopardising the human–dog bond should not be underestimated by simple, broad-scale policies.

Data on dogs castrated for behavioural reasons were excluded from the current analysis to avoid sampling bias. We also excluded data from dogs where the veterinarian recommended castration in case such advice had given in the light of reports of unwelcome behavioural traits. The current data reveal that lower AAC or PLGH is associated with the emergence (or persistence) of many unwelcome behaviours and that while odds ratios appear close to 1 on a small scale of a percentage or a week, these odds accumulate to substantial differences over time, parallelling the clinical differences observed between entire and castrated dogs. They also show that only urine marking and howling when left alone can be expected to be less prevalent as a result of castration. It is important to note that the population control benefits of castration may continue to trump the risks of undesirable behaviour. However, veterinarians have historically recommended castration for behavioural traits, such as boisterousness, that appear unrelated to gonadal hormones. The current data may serve to question this practice.

The current data indicate that some forms of aggression, a category of response intimately connected to fear [41], is significantly and positively associated with lower AAC and PLGH. This aligns with previous evidence that shyness (as opposed to boldness) is higher in castrated dogs [8]. It is possible that, during the transition through puberty, sex hormones play a role in proofing dogs against fearfulness in later life. That said, the value of comprehensive, appropriate socialisation of pups (especially between 3 and 14 weeks of age) as a means of reducing fear and aggression cannot be overstated [12].

The reduction in sex hormones during puberty associated with early castration may affect the coping strategies dogs tend to adopt. A proactive coping style can be considered a higher tendency towards both actively engaging threats with aggressive behaviours, and also actively avoiding threats where that option may serve better [42]. Higher scores in both fear and aggression may indicate dogs that are either experiencing more acute fear, or coping with similar levels of fear in a more overt and impulsive way.

Beyond the timing of castration, it is worth considering why some owners decide not to castrate their dogs. It may be that those who do not gonadectomise their dogs represent two different ownership styles: those who ignore gonadectomy campaigns and those with the skills to manage entire dogs. The former may be less responsible than the latter and may have influenced the current results by failing to remediate unwelcome behaviours as they emerged. If this were the case, the effects of castration may be even more extreme than our results suggest, as the lower association of entire dogs with problem behaviours may be further offset by problem behaviours in the population of entire dogs that have their genesis in environment and management more than in castration.

Studies using data from C-BARQ are sometimes questioned because they may reflect some owner bias but C-BARQ results have been independently validated by comparison with the results of observational assessments (e.g., refs [43] and [44]). We acknowledge that respondents generally need to seek out C-BARQ to participate in the survey, so they are perhaps more likely to be from among those skilled owners who manage entire dogs successfully than from less responsible owners with potentially less interest in their dog’s behaviour. We also acknowledge that the current study has not dealt with potential differences in the effects of castration before and after puberty. This would require validated information on the age at puberty of each individual. Such an approach may enhance breed-specific studies that unpack the role of breed differences, such as in reactivity, fearfulness, excessive barking and aggressive behaviours reported in small-dog breeds compared to larger dogs.

Aggressive behaviour has been identified as more common in male dogs in several studies [2, 3, 4, 6], and dog bites/attacks have been attributed heavily to male dogs, accounting for 70% [45], of bites in some studies. As such, current evidence from this study and others [46] on the behavioural effects of castration on male dogs may seem contradictory. Perhaps the earlier studies included more intact males and perhaps the decrease in dog-related aggression in the current findings, in part at least, reflects the fact that entire dogs are now so rare in the US (where C-BARQ data come from) that male–male competition for intact females is rarely seen.

Perhaps the decrease in dog-related aggression in the current findings, in part at least, reflects the fact that entire dogs are now so rare in most parts of the developed world (where C-BARQ data come from) that male–male competition for intact females is rarely seen.

On a related note, opposition to castration male dogs in some countries is may be based on the belief that castrated animals will be more fearful and therefore less useful as guard dogs [47]. Interestingly, the current results suggest that this perception is both true and false: castrated dogs are more fearful, but also more aggressive towards strangers and bark more. As such, current findings could have important implications for dog-population management.

The 2 undesirable behaviours that arose with increased PLGH were indoor-marking and howling when left alone. The association between testosterone and the motivation to mark with urine has clear biological advantages, since urine can alert female dogs to the presence of an entire male and thus increase the chances of reproduction. That said, it is more likely that this type of urine marking is directed at other (entire) male dogs. Indoor marking overcomes house-training so may reflect a particularly strong motivation to mark. Surprisingly, increased PLGH was also associated with less mounting but it should be noted that the C-BARQ question about mounting focuses on mounting of objects including furniture and people. Perhaps this activity is motivated by play or anxiety and that mounting by entire dogs is chiefly directed to dogs. Meanwhile, howling when left alone is a feature of SRD [17]. The role of vocalization in SRD may be to reunite the social group, but why this should be a task for males is less clearly understood. Notably, increased PLGH was associated with less barking when excited or alarmed and less excitement when doorbells rang. This presents a picture of entire male dogs perhaps being both less excitable and less prone to fear, yet more prone to SRD. The two are not necessarily opposed, particularly when we consider that the increased use of scent marking by entire male dogs may point to increased social interest. Meanwhile, trainability in recall and retrieving decreased with PLGH. This may reflect distraction by the social signals available from other dogs’ scents in entire dogs and retention of playfulness in castrated dogs. Natural puppy playfulness may decrease after puberty and this may explain why entire dogs are less excitable.

Increased food stealing was significantly related to AAC (but not to PLGH) perhaps reflecting an effect of owner-mediated dietary restriction after castration. Meanwhile, coprophagia was significantly related to PLGH (but not to AAC). We note that coprophagia may target the faeces of various species and may have heterogeneous causes. Rolling in faeces may be an exploratory behaviour that dogs grow out of, if left entire.

There are important limitations to the study which must be noted. In particular, caution with regard to assuming a causal association between castration and problematic behaviours. While attempts were made to exclude animals that were castrated because of problematic behaviour, it is possible that some owners misremembered or incompletely reported the reasons behind their decision to castrate. Equally, there may be limitations regarding the applicability of the survey result to the companion dog population as a whole because people who sought out and completed the C-BARQ survey may be more engaged with issues surrounding problematic behaviours generally and may also have different views about the timing of castration when compared with companion dog owners as a whole.

There are also limitations surrounding the PLGH metric which need to be considered. While we considered it important to refer to a metric that takes into account the amount of time which a dog has had to experience of behavioural tendencies developed under the influence of gonadal hormones, it is important to understand that dogs with the same PLGH could have wildly different ages. While we attempted to take this into account by including the age of CBARQ evaluation in our models, this limitation should be kept in mind. A further limitation is that the current data come from a wide range of breeds that mature skeletally, socially and sexually at different ages. This means that it, in many cases, we cannot be sure whether castration occurred before, during or after puberty. Future researchers may wish to approach the same or similar behavioural data-sets with the benefit of breed-specific data on the usual timing of puberty.

While both AAC and PLGH were of interest in this analysis, AAC may be more suitable for understanding behaviours that develop with sexual maturity and, once learned, are relatively resistant to extinction. As PLGH captures how long the dog has lived with gonadal hormones (after gonadectomy at any age), it may be more suitable for understanding behaviours that, once learned, are more amenable to extinction and so potentially modifiable. The interplay between these two variables may merit further scrutiny since the subtleties of these interactions may enhance veterinary advice on castration, especially in the case of dogs predisposed to certain behavioural tendencies.

## Supporting information

S1 Table Numbers of dogs of the 27 breeds and crosses tested in the current study. (DOCX)

S2 Table C-BARQ attributes showing percentage differences of high C-BARQ item scores between entire and castrated male dogs. A negative valence in the % difference column indicates that entire dogs show high levels of the behaviour less frequently than castrated dogs.
(DOCX)

S1 Fig The distribution of lifetime exposure to gonadal hormones in the castrated male dogs (n = 6235) in the current study. (DOCX)
