# Supplementary material for: Behavioural risks in male dogs with minimal lifetime exposure to gonadal hormones may complicate population-control benefits of desexing
Source: PLoS One. 2018 May 2;13(5):e0196284. doi: 10.1371/journal.pone.0196284 (PMC5931473; doi:10.1371/journal.pone.0196284)
Supplement: S1 Table — (DOCX) [file pone.0196284.s001.docx]

**S1 Table. Numbers of dogs of the 27 breeds and crosses tested in the current study**

| **Breed reported** | **Number in current study** |
| --- | --- |
| Mixed Breed/Unknown | 1959 |
| Labrador Retriever | 358 |
| German Shepherd | 183 |
| Golden Retriever | 178 |
| Border Collie | 168 |
| Australian Shepherd | 151 |
| American Pit Bull Terrier | 107 |
| Poodle (Standard) | 95 |
| Australian Cattle Dog | 93 |
| Rottweiler | 84 |
| Jack Russell Terrier | 79 |
| Chihuahua | 76 |
| Soft Coated Wheaten Terrier | 76 |
| Shetland Sheepdog | 75 |
| Boxer | 70 |
| Cocker Spaniel (American) | 70 |
| Beagle | 68 |
| Doberman Pinscher | 65 |
| Dachshund | 61 |
| Shih Tzu | 43 |
| Greyhound | 55 |
| Siberian Husky | 55 |
| Bernese Mountain Dog | 54 |
| Collie | 50 |
| Pit Bull mix | 50 |
| Miniature Schnauzer | 45 |
| Shiba Inu | 56 |
