# Supplementary material for: Behavioural risks in male dogs with minimal lifetime exposure to gonadal hormones may complicate population-control benefits of desexing
Source: PLoS One. 2018 May 2;13(5):e0196284. doi: 10.1371/journal.pone.0196284 (PMC5931473; doi:10.1371/journal.pone.0196284)
Supplement: S2 Table — A negative valence in the % difference column indicates that entire dogs show high levels of the behaviour less frequently than castrated dogs. (DOCX) [file pone.0196284.s002.docx]

S2 Table. C-BARQ attributes showing percentage differences of high C-BARQ item scores between entire and castrated male dogs. A negative valence in the % difference column indicates that entire dogs show high levels of the behaviour less frequently than castrated dogs.

| Percentage Difference between entire dogs and castrated dogs | C-BARQ section | Question |
| --- | --- | --- |
| 6.53 | Trainability | When off the leash, returns immediately when called |
| 7.66 | Trainability | Will “fetch” or attempt to fetch sticks, balls, or objects |
| -5.04 | Aggression | When approached directly by an unfamiliar **adult** while being walked/exercised on a leash |
| -5.87 | Aggression | When an unfamiliar person approaches you or another member of your family at home |
| -8.54 | Aggression | When mailmen or other delivery workers approach your home |
| -6.88 | Aggression | When strangers walk past your home while your dog is outside or in the yard |
| -7.85 | Aggression | When joggers, cyclists, roller-bladers or skateboarders pass your home while your dog is outside or in the yard |
| -10.98 | Aggression | When approached directly by an unfamiliar **female** dog while being walked/exercised on a leash |
| -5.41 | Aggression | Toward unfamiliar persons visiting your home |
| -6.76 | Predation | Toward cats, squirrels or other small animals entering your yard |
| -5.81 | Predation | Chases or would chase squirrels, rabbits and other small animals given the opportunity |
| -6.66 | Fear and Anxiety | When approached directly by an unfamiliar **adult** while away from your home |
| -6.61 | Fear and Anxiety | When approached directly by an unfamiliar **child** while away from your home |
| -11.49 | Fear and Anxiety | In response to sudden or loud noises (e.g., vacuum cleaner, car backfiring, road drills, objects being dropped, etc.) |
| -7.92 | Fear and Anxiety | When unfamiliar persons visit your home |
| -5.40 | Fear and Anxiety | When an unfamiliar person tries to touch or pet the dog |
| -5.13 | Fear and Anxiety | In response to strange or unfamiliar objects on or near the sidewalk (e.g., plastic trash bags, leaves, litter, flags flapping, etc. |
| -9.39 | Fear and Anxiety | When examined/treated by a veterinarian |
| -12.31 | Fear and Anxiety | During thunderstorms, firework displays, or similar events |
| -6.93 | Fear and Anxiety | When approached directly by an unfamiliar dog of the same or larger size |
| -7.37 | Fear and Anxiety | When approached directly by an unfamiliar dog of a smaller size |
| -7.13 | Fear and Anxiety | When first exposed to unfamiliar situations (e.g., first car trip, first time in elevator, first visit to veterinarian, etc.) |
| -6.42 | Fear and Anxiety | In response to wind or wind-blown objects |
| -12.05 | Fear and Anxiety | When having nails clipped by a household member |
| -8.91 | Fear and Anxiety | When groomed or bathed by a household member |
| -5.66 | Fear and Anxiety | When having his/her feet toweled by a household member |
| -6.80 | Fear and Anxiety | When unfamiliar dogs visit your home |
| -10.43 | Fear and Anxiety | When barked at, growled at, or lunged at by an unfamiliar dog |
| 5.94 | Separation related behaviour | Howling |
| -5.04 | Excitability | When doorbell rings |
| -5.77 | Excitability | Just before being taken on a car trip |
| 7.42 | Energy level | Playful, puppyish, boisterous |
| 9.69 | Energy level | Active, energetic, always on the go |
| -6.02 | Miscellaneous | Rolls in animal droppings or other “smelly” substances |
| -6.96 | Miscellaneous | Eats own or other animals’ droppings or feces |
| 7.64 | Miscellaneous | Mounts’ objects, furniture, or people |
| -6.62 | Miscellaneous | Steals food |
| 8.68 | Miscellaneous | Urinates against objects/furnishings in your home |
| -6.42 | Miscellaneous | Barks persistently when alarmed or excited |
| -7.27 | Miscellaneous | Licks him/herself excessively |
