# Supplementary material for: Behavioural risks in male dogs with minimal lifetime exposure to gonadal hormones may complicate population-control benefits of desexing
Source: PLoS One. 2018 May 2;13(5):e0196284. doi: 10.1371/journal.pone.0196284 (PMC5931473; doi:10.1371/journal.pone.0196284)
Supplement: S1 Fig — (DOCX) [file pone.0196284.s003.docx]

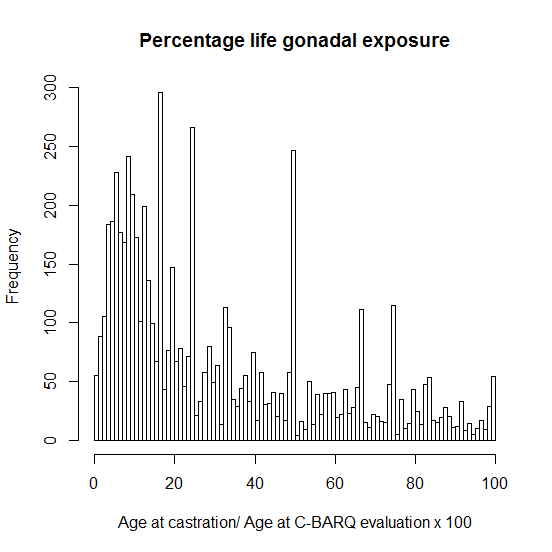


**S1 Fig. The distribution of lifetime exposure to gonadal hormones in the castrated male dogs (n = 6235) in the current study.**
